# The efficacy of self-monitoring of blood glucose (SMBG) intervention package through a subscription model among type-2 diabetes mellitus in Malaysia: a preliminary trial

**DOI:** 10.1186/s13098-024-01379-9

**Published:** 2024-06-21

**Authors:** Sa’ida Munira Johari, Nurul Huda Razalli, Kai Jia Chua, Suzana Shahar

**Affiliations:** 1 Alpro Pharmacy SDN. BHD., Seremban, Malaysia; 2 Dietetic Program, Centre for Healthy Aging and Wellness (H-CARE), Faculty of Health Sciences, Universiti Kebangsaan Malaysia, Jalan Raja Muda Abdul Aziz, 50300 Kuala Lumpur, Malaysia

## Abstract

**Background:**

The aim of this study was to determine the effect of a Self-Monitoring Blood Glucose (SMBG) intervention package through a subscription model in improving HbA1c and health parameters among type-2 diabetes mellitus (T2DM) individuals in Malaysia.

**Methods:**

This is a quasi-experimental study involving a total number of 111 individuals with T2DM (mean age 57.0 ± 11.7 years, 61% men) who were assigned to intervention (n = 51) and control (n = 60) groups. The intervention group participants were the subscribers of SugO365 program which provided a personalized care service based on self-recorded blood glucose values. Subscribers received a Contour^®^ Plus One glucometer which can connect to Health2Sync mobile app to capture all blood glucose readings as well as physical and virtual follow up with dietitians, nutritionists, and pharmacists for 6 months. Outcome measures were body weight, body mass index (BMI), random blood glucose (RBG), glycated haemoglobin (HbA1c) and health-related quality of life (HRQoL, assessed by SF-36 questionnaire). Data were measured at baseline, third and sixth months.

**Results:**

Repeated-measure analysis of covariance showed significant improvement in HbA1c level (ƞp^2^ = 0.045, p = 0.008) in the intervention (baseline mean 7.7% ± 1.1%; end mean 7.3% ± 1.3%) as compared to control (baseline mean 7.7% ± 0.9%; end mean 8.1% ± 1.6%) group. Similar trend was observed for Role Emotional domain of the quality of life (ƞp^2^ = 0.047, p = 0.023) in the intervention (baseline mean 62.8 ± 35.1, end mean 86.3 ± 21.3) compared to control (baseline mean group 70.5 ± 33.8; end mean 78.4 ± 27.3) group. Negative association was found in HbA1c changes using Z-score and Physical Function domain (r = − 0.217, p = 0.022).

**Conclusion:**

A 6 months SMBG intervention package through a subscription model improved blood glucose control as measured by HbA1c and health-related quality of life, particularly the Role Emotional domain. Elevated HbA1c levels are correlated with decreased physical function.There is a need to further examine the efficacy of SMBG intervention package using a larger sample and a longer period of intervention and to determine its cost efficacy.

## Introduction

Type 2 Diabetes Mellitus (T2DM) stands out among the most prevalent chronic conditions globally as well as Malaysian public health concern. The estimation of the current and future burden of T2DM is important in-order to allocate community and health resources. In 2015, the National Health Morbidity Survey (NHMS) reported 17.5% prevalence of T2DM and recently, the prevalence continues to rise to 18.3% (NHMS, [44]) among adults in Malaysia. This affects about 3.3 million adults in our country (IDF Atlas). Moreover, Shaw, et al. [56] reported that Malaysia was predicted to be in the list of top ten country (out of 91 countries) with the highest prevalence of diabetes in 2030. Globally, the World Health Organization (WHO) projects T2DM as the seventh leading cause of death and estimates that there will be 366 million adults with T2DM in 2030 [52].

Education-based interventions for T2DM have been implemented and studied extensively [39] and [3]). Specific lifestyle intervention programs, proven effective in decreasing the occurrence and management of T2DM, necessitate a multifaceted approach for sustained control [15, 19] and [32]). For example, in an intensive lifestyle intervention study for T2DM by Johansen, et al. [26], a reduction in glucose-lowering medications occurred in 47 participants (73.5%) in the intervention group as compared to only 9 participants (26.4%) in the control group [95% CI, 28.6–65.3]. Another study also found out that lifestyle intervention significantly reduced more weight than the participants in control group with net difference of − 7.9% [95% CI, − 8.3% to − 7.6%] [17].

Various lifestyle interventions for T2DM have also proven to improve HbA1c (Yang, et al. [62]). HbA1c which indicates glycaemic control is a significant determinant for risk of diabetes complications and mortality [50]. This finding leads to the usage of HbA1c to monitor long-term glycaemic control and to guide therapy for diabetic patients [13]. Glycaemic control is dynamic, changing over the natural history of diabetes (Walraven, et al., [60]). Patterns with consistently high indicates higher prevalence for complications as well as mortality [46]. Based on previous research, the progression of T2DM, as measured by HbA1c is well-regulated with better diabetes knowledge and self-care empowerment [6].

Self-care includes several key activities such as healthy eating, regular exercise, medication adherence, foot care, smoking cessation, and self-monitoring of blood glucose (SMBG) (Goh, et al., [16]). Although self-care is crucial in the management of T2DM progression, this lifestyle is considered lacking among T2DM patients due to lack of motivation and difficulties in changing habits [37]. SMBG in developing countries is considered a major healthcare challenge where patients often have sub-optimal affordability for frequent blood glucose testing [27]. Specifically, In Asia, only 29.7% of T2DM patients are estimated reporting regular SMBG usage [9]. Thus, there is a need to determine its effectiveness in this population.

In 2021, a retrospective cohort study in Taiwan reported that SMBG positively associated with better blood glycaemic control [57]. An insight into SMBG within the Asian population can provide valuable data due to the diverse factors influencing the effectiveness of an intervention across different population [65]. Additionally, a subgroup analysis showed that using SMBG to adjust therapy contributed significantly to the reduction of HbA1c and no significant improvement was shown without therapy adjustment [7]. This indicates the importance of structured SMBG to modify blood glucose readings for rectifying disease condition and slower the progression of T2DM.

Aside from that, T2DM has dynamic impact on health-related quality of life (HRQoL). Diabetes is linked to various complications and patient attitudes, which together negatively impact multiple dimensions of HRQoL (Pan, et al., [48]). The disease itself can reduce work productivity and contributes to health-related limitations, especially for those patients with poorly control glucose level [22]. The proposed mechanism is the improvement in glycaemic control after implementing SMBG may be in favour of physical and emotional functioning which may improve daily activities. SMBG provides real-time feedback to diabetic patients about their glycaemic control [9]. Diabetic patients treated with insulin rely on SMBG for guidance to adjust insulin doses in achieving desired glucose level without hypoglycaemia (Mbanya, et al., [34]). On the other hand, among T2DM patients without insulin, SMBG can also promote self-management and improvements in glycated haemoglobin (HbA1c) (Farmer, et al., [14]).

SMBG provides valuable data that healthcare providers can use to tailor treatment plans to meet individual patients’ needs. Subscription models can provide a sustainable approach ensuring continuous support and resources for managing T2DM. Despite these benefits, structured SMBG practices are not widely accessible to T2DM patients. Additionally, there is a lack of research specifically targeting T2DM patients in the Asian region, particularly studies examining the efficacy of SMBG. To address this gap, this study aims to assess the effectiveness of an SMBG intervention package delivered via a subscription model at a community pharmacy in Malaysia. The study will focus on enhancing blood glucose control (HbA1c) and improving the health-related quality of life among adults diagnosed with T2DM.

## Methodology

### Study design, sampling

This is a preliminary quasi experimental trial to determine the feasibility and effectiveness of SMBG intervention package through a subscription model among 111 participants with T2DM. Study participants were recruited from Alpro Pharmacy clients across Malaysia who were diagnosed with T2DM. Participants were recruited using convenient sampling from June 2020 until July 2022. Inclusion criteria were T2DM patients with uncontrolled blood glucose level for the past 6 months (defined as HbA1c ≥ 6.3%) [36]. Other than that, participants must be 18 years old and above with no known terminal illness or mental disturbance and ability to communicate in Malay, English, or Chinese. For intervention group, the subscription was not sponsored; only those who subscribed to any package (Appendix A) for at least continuous 6 months duration and met the inclusion criteria joined the intervention group. Recruitment and data collection were conducted from June 2020 to July 2022 by trained enumerators, nutritionists, dietitians, and pharmacists from Alpro Pharmacy.

### Data collection

Data was gathered using a bilingual questionnaire form that comprises three sections. The first section covers socio-demographic information, the second section includes anthropometry and biomarkers, and the third section features the SF-36 questionnaire. After recruitment, data was collected at baseline, the 3rd month, and the 6th months.

### Intervention implementation

#### Intervention group

SugO365 is a subscription program where participants received a Contour^®^ Plus One glucometer which can connect to Health2Sync mobile app. In this subscription program, participants received glucose strips supply along the subscription period. Participants were instructed on how to use the glucometer at home for self-monitoring of blood glucose (SMBG) upon subscribing. For the first week of subscription, participants need to do SMBG following the monitoring template (Table 1) and synchronize the glucose readings from the glucometer to the mobile app at least twice a day throughout the intervention duration. Afterwards, nutritionists and dietitians in-charge will receive the readings from the app on Health2Sync dashboard and will provide online education from time to time based on the blood glucose readings. The online education sessions were given through the mobile app, as well as phone calls when it is necessary. For example, if the participant was hypoglycaemic, the online consultation will discuss the management of hypoglycaemia. Meanwhile, upon refilling the strips and during data collection days, the participants received one-to-one education advice for diabetic management with nutritionists or dietitians in the pharmacy outlets. Content of the education sessions were based on topics related to T2DM booklet (Appendix B) and the topics are described in Table 2. The participants in the intervention group will also join webinar sessions on diabetic management topics every 3 monthly. The topics were listed in Table 3.

**Table 1:** SMBG monitoring template for the first 7 days

Day	Pre BF	2 h post BF	Pre L	2 h post L	Pre D	2 h post D	Pre bed
1	X	X					
2			X	X			
3					X	X	
4	X						X
5		X	X				
6				X	X		
7						X	X

*BF* breakfast, *L* lunch, *D* dinner, *X* SMBG

**Table 2:** T2DM booklet content and education schedule for the intervention group

Month	Education session content
1	Understanding T2DM and blood glucose monitoring - Insulin: roles, resistance and shortage - T2DM risk factors, symptoms and complications - How is blood glucose tested?
2	Hyperglycaemic and hypoglycaemic symptoms - What are hyper and hypoglycaemia? - My healthy plate - Meal time, total and types of carbohydrate
3	Tips when eating out - Hidden sugar and healthy snack choices - Hawker hacks, watch out your calories
4	Lifestyle modification for blood glucose control - Lifestyle choice to avoid - Simple physical activity and principle of exercise
5	The importance of footcare - Footcare practice for diabetics - Proper foot care and annual screening
6	Tips when travelling - Travel plan when you have T2DM - See a doctor, pack your medicines

**Table 3:** Webinar session schedule and topic

Month	Topic
3	Nutrition label and carbohydrate counting
6	Why is my blood sugar level always high?

#### Control group

The control group were recruited among patients matched with intervention group and received regular offline education session at the pharmacy outlets they attended. Initially, body weight, blood glucose, and HbA1c were assessed, and diabetes education was provided, covering topics related to complications and the importance of management. Dietary counselling focused on balanced nutrition and a diabetic diet were also provided. Scheduled appointments for monitoring and follow-up were arranged every 3 months, with data collection carried out at the same time.

#### Compliance

Compliance in the intervention group was assessed by attendance at scheduled appointments (during glucose strip refill and data collection), adherence to SMBG, completion of education sessions, both online and offline and adherence to medication prescribed. The Health2Sync app was utilized to monitor SMBG, attendance scheduled follow up, education sessions and medication adherence were recorded. Subjects having greater than 75% points are considered in the good compliance group.

### Measures

The outcome measures included in this study were body weight, body mass index (BMI), random blood glucose (RBG), glycated haemoglobin (HbA1c) and Health-related quality of life (HRQoL). Body weight and BMI were measured using TANITA body scale (Tanita Corp, USA). RBG and HbA1c were measured using Contour^®^ Plus One Glucometer (Ascencia, Switzerland) and Cobas b 101 (Roche, Switzerland) machine respectively. HRQoL was measured by SF-36 questionnaire (Instrument Ware & Sherbourne, [24]; [53]) which consists of eight scales that produce two summary measures: Physical Health and Mental Health. The physical health measure includes four scales: Physical Functioning (10 items), Role-Physical (4 items), Bodily Pain (2 items), and General Health (5 items). The Mental Health measure comprises four scales: Vitality (4 items), Social Functioning (2 items), Role-Emotional (3 items), and Mental Health (5 items). There is an additional item called self-reported health transition, which needed to be answered by the respondent but not included in the scoring process. It also uses Likert scales and yes/no options to assess function and well-being across the 36 items. Scoring the SF-36 involves standardizing the algorithm to obtain scores ranging from 0 to 100, with higher scores indicating better health status.

### Statistical analysis

Descriptive and chi-square analyses were performed on categorical data. Independent Student t-test, Mann–Whitney U test, repeated measure analysis of covariance (ANCOVA) was employed to evaluate the effects of the intervention on parameters. All analyses were performed using IBM SPSS Statistics version 28. In these analyses, outcome measures were sociodemographic data (age, gender, race, education status, household income, occupation, and type of treatment), anthropometry data (weight, height and body mass index, BMI) and biomarkers (random blood glucose and HbA1c). Meanwhile, outcome measures for HRQoL were Physical Health domain (Physical Functioning, Role-Physical, Bodily Pain, and General Health) and Mental Health domain (Vitality, Social Functioning, Role-Emotional, and Mental Health).

### Ethical

Ethical approval was obtained from Universiti Kebangsaan Malaysia Medical Centre (UKMMC) Ethical Committee (UKM PPI/111/8/JEP-2021-618). Informed consent was obtained from all participants.

## Results

### Sociodemographic characteristics

**Fig. 1:**
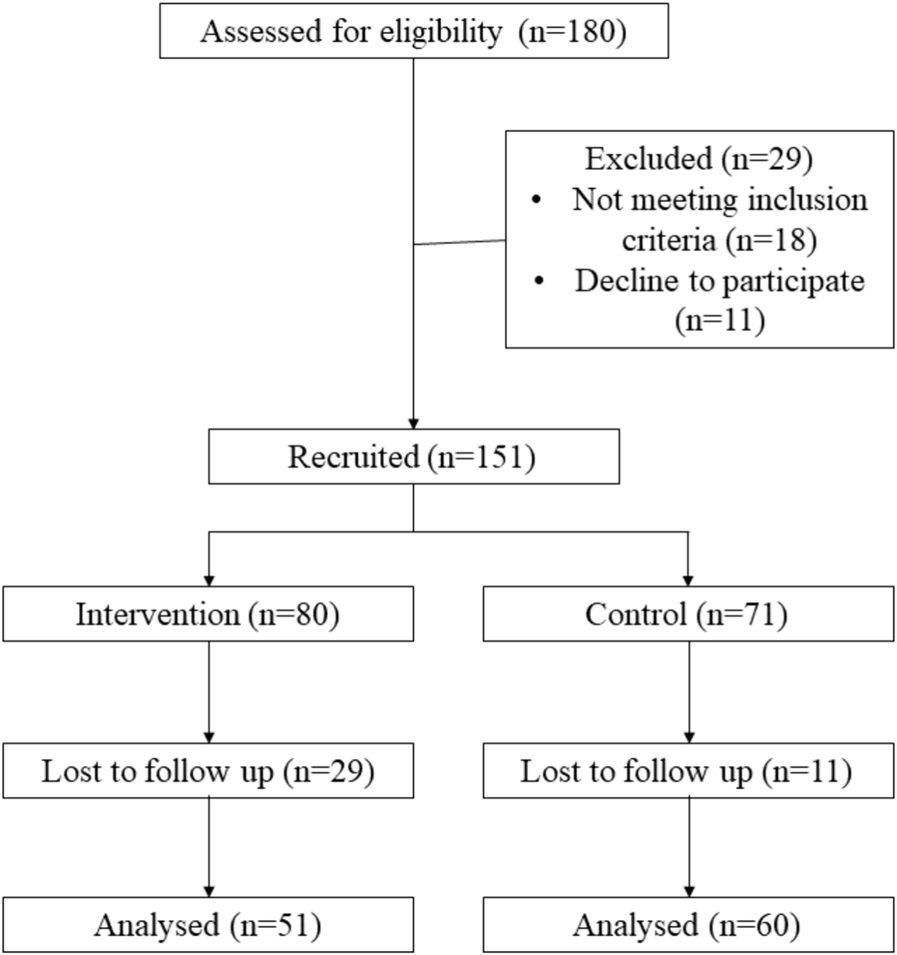
Consort flow chart for intervention trial

Overall completion rate of the intervention was 73.5% (n = 111 completed the study out of n = 151 recruited); 36.3% and 15.5% dropout rates in the intervention group and control group respectively (Fig. 1). The intervention group adherence rate was 63.8% while in the control group was 84.5%. As shown in Table 4, the age of the participants ranged from 33 to 81 years; the mean age was 57.0 ± 11.7 years. Of the participants, 61% (n = 68) were men. Percentage of Malays and Chinese participants were about the same which were 44.1% (n = 49) and 42.3% (n = 47) respectively. Both groups were comparable with respect to mean age, gender, race, education status, occupation, and type of treatment. However, the result shows a larger number of higher household income among participants in the intervention group (p < 0.05). Accordingly, this variable was used as covariate in repeated measure ANCOVA.

**Table 4:** Sociodemographic and health characteristics of subjects (n = 111)

Sociodemography	Profile			
	Intervention (n = 51)	Control (n = 60)	Total (n = 111)	Significant level
Age (mean years ± sd)	58.2 ± 12.9	56.2 ± 10.7	57.0 ± 11.7	0.389
Gender [n (%)]				
Men	27 (52.9)	41 (68.3)	68 (61.3)	0.097
Women	24 (47.1)	19 (31.7)	43 (38.7)	
Race [n (%)]				
Malay	19 (37.3)	30 (50.0)	49 (44.1)	0.107
Chinese	27 (52.9)	20 (33.3)	47 (42.3)	
Indian	5 (9.8)	10 (16.7)	15 (13.5)	
Education status [n (%)]				
No formal education	12 (23.5)	10 (16.7)	22 (19.8)	0.366
Formal education	39 (76.5)	50 (83.3)	89 (80.2)	
Household income [n (%)]				
≤ RM2500	16 (31.4)	34 (56.7)	50 (45.0)	0.008*
> RM2500	35 (68.6)	26 (43.3)	61 (55.0)	
Occupation [n (%)]				
Unemployed/Retired	20 (39.2)	32 (53.3)	52 (46.8)	0.137
Employed/Self-Employed	31 (60.8)	28 (46.7)	59 (53.2)	
Treatment [n (%)]				
Insulin	28 (54.9)	24 (40.0)	52 (46.8)	0.117
Oral hypoglycaemic agent	23 (45.1)	36 (60.0)	59 (53.2)	

^*^p < 0.05 significant difference between groups using chi-square test

### Anthropometry and biomarkers

**Fig. 2:**
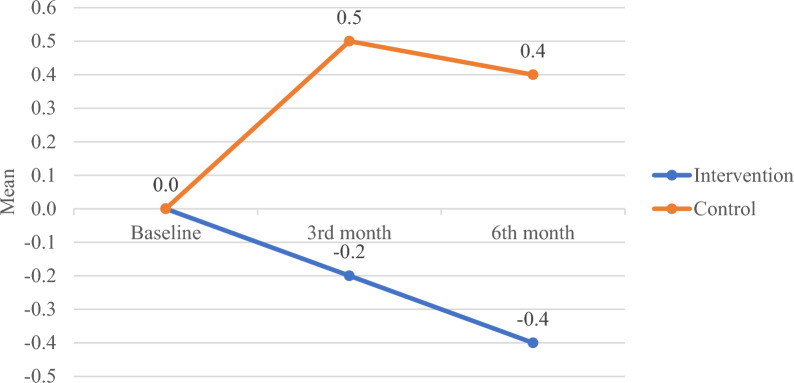
Mean changes of HbA1c (3rd and 6th month minus baseline)

Repeated measure ANCOVA showed that the intervention group had significantly improved HbA1c level across the 6 months data collection period (Table 5). During the 3 month, the intervention group showed improvement (mean changes − 0.2) in HbA1c level and greater improvement (mean changes − 0.4) at 6th month as compared to the control group (mean changes + 0.5 and + 0.4 respectively) (Fig. 2).

**Table 5:** Anthropometry and biomarker values at baseline, 3rd month and 6th month follow ups (presented as mean ± SD)

	Intervention group (n = 51)				Control group (n = 60)			Repeated measures		
	Baseline	3rd month	6th month		Baseline	3rd month	6th month	Interaction effect, p (ƞp^2^)	Time effect, p (ƞp^2^)	Group effect, p (ƞp^2^)
*Anthropometry*										
Weight (kg)	71.0 ± 13.6	69.8 ± 14.0	70.2 ± 13.9		73.8 ± 16.5	73.9 ± 17.0	74.0 ± 17.0	0.851 (0.002)	0.363 (0.013)	0.458 (0.010)
Body mass index (kg/m^2^)	27.0 ± 5.0	26.5 ± 5.1	26.7 ± 5.1		27.4 ± 4.9	27.5 ± 5.0	27.5 ± 5.0	0.537 (0.006)	0.085 (0.023)	0.161 (0.017)
*Biomarker*										
Random blood glucose (mmol/L)	9.4 ± 3.6	8.3 ± 2.6	8.3 ± 2.2		10.5 ± 4.7	10.6 ± 4.6	9.8 ± 3.8	0.235 (0.013)	0.063 (0.025)	0.825 (0.002)
HbA1c (%)	7.7 ± 1.1	7.5 ± 1.3	7.3 ± 1.3		7.7 ± 0.9	8.2 ± 1.8	8.1 ± 1.6	0.008* (0.045)	0.296 (0.011)	0.509 (0.006)

*p < 0.05, repeated measured ANCOVA, controlled for household income

### Health-related quality of life

Analysis of HRQoL score revealed a significant interaction effect in one of Mental Health scale, the Role-Emotional (Table 6). This parameter marked a significant score improvement of 37.4% among the intervention group as compared to 11.2% among the control group.

**Table 6:** Health-related quality of life parameter values at baseline, 3rd month and 6th month follow ups (presented as mean % ± SD)

	Intervention (n = 51)		Control (n = 60)		Repeated measures		
	Baseline	6th month	Baseline	6th month	Interaction effect, p (ƞp^2^)	Time effect, p (ƞp^2^)	Group effect, p (ƞp^2^)
Physical health							
Physical functioning	73.8 ± 13.9	71.2 ± 16.9	76.8 ± 19.4	69.5 ± 13.7	0.184 (0.016)	0.025* (0.046)	0.838 (0.000)
Role-physical	56.4 ± 33.9	86.3 ± 18.2	65.4 ± 37.4	83.3 ± 25.1	0.241 (0.013)	0.001** (0.122)	0.790 (0.001)
Bodily pain	67.6 ± 16.4	66.2 ± 19.5	67.7 ± 20.5	66.8 ± 15.1	0.997 (0.000)	0.710 (0.001)	0.827 (0.000)
General health	67.1 ± 67.1	74.3 ± 10.9	60.5 ± 12.9	68.8 ± 10.3	0.114 (0.023)	0.012* (0.057)	0.001** (0.104)
Overall physical health	64.7 ± 10.6	72.8 ± 10.0	66.4 ± 12.5	70.7 ± 9.7	0.078 (0.029)	0.007** (0.065)	0.684 (0.002)
Mental health							
Vitality	58.9 ± 14.2	66.3 ± 13.6	62.2 ± 13.9	65.1 ± 11.2	0.125 (0.022)	0.030* (0.043)	0.755 (0.001)
Social functioning	80.6 ± 15	76.4 ± 14.1	78.3 ± 16.6	79.4 ± 13.3	0.366 (0.008)	0.068 (0.031)	0.655 (0.002)
Role-emotional	62.8 ± 35.1	86.3 ± 21.3	70.5 ± 33.8	78.4 ± 27.3	0.023* (0.047)	0.031* (0.042)	0.823 (0.000)
Mental health	76.3 ± 12.5	81.0 ± 9.70	74.0 ± 14.5	81.3 ± 8.2	0.370 (0.007)	0.002** (0.087)	0.650 (0.002)
Overall mental health	69.1 ± 12.2	76.8 ± 9.6	69.1 ± 12.6	74.6 ± 9.0	0.196 (0.015)	0.009** (0.062)	0.639 (0.002)

*p < 0.05, **p < 0.01 repeated measures ANCOVA, controlled for household income

This study also found significant time effect in parameters namely Physical Functioning, Role Physical, General Health, and Overall Physical Health. Likewise, in Mental Health domain, significant time effect was found in Vitality, Role Emotional and Mental Health, resulting in significant Overall Mental Health value. Generally, the value of all significant parameters showed positive changes in both groups, except Physical Functioning. Meanwhile, General Health parameter showed significant group effect, however mean changes was higher in control group (+ 8.3%) compared to intervention group (+ 7.2%).

### Correlation between HbA1c and health-related quality of life

In-order to measure the relationship between blood glucose control and HRQoL, a nonparametric Spearman’s correlation was employed. As shown in Table 7, there is a significant negative relationship between HbA1c and Physical Functioning [r_s_ = − 0.217 (p < 0.05)].

**Table 7:** Relationship of HRQoL scale score (6th month minus baseline) using Z-score and HbA1c changes using Spearman’s correlation

Δ health-related quality of life	r	p
Physical health		
Physical functioning	− 0.217	0.022*
Role-physical	0.030	0.756
Bodily pain	− 0.079	0.410
General health	0.032	0.740
* Overall physical health*	0.018	0.852
Mental health		
Vitality	0.058	0.545
Social functioning	0.101	0.290
Role-emotional	0.026	0.783
Mental health	0.034	0.721
* Overall mental health*	0.062	0.517

*p < 0.05 using Spearman's correlation

## Discussion

From the present study, the completion rate was 73.5%. In-order to carry out research in a retail setting, dietitian, nutritionist, and pharmacist are well suited to hold expanded role in the healthcare system (Mossialos, et al., [40]), however, the adherence to follow up sessions is a challenge, and it can be the cause of participants withdrawing from the study. In some cases, the participants, who were also the consumers in retail setting do not have the urgency for treatment plan thus causing the decrease in the adherence rate. Survey form is preferred for better adherence from consumers for a retail pharmacy to conduct a research-based program as suggested by Schuessler, et al,. [54] and we also acknowledged this following completion of this study.

Nevertheless, this study has successfully documented that such intervention trial combining health devices monitoring and subscription plan as well as consultation in hybrid approach, i.e. online and face to face is feasible to be conducted in a retail pharmacy. The advancement of technology plays a significant role in conveying diet and health education to promote SMBG. Connected health devices and telemonitoring have the potential to better support T2DM care management goals [35].

The subscription program was intended for those with uncontrolled T2DM, and this study's evidence shows that the intervention package has benefits beyond self-motivation for people with higher income. In Asia Pacific region, although complex issues of cost and affordability remain, the efficacy of this purposeful tool to tailor management plan of this disease is undeniable (Chowdhury, et al,. [9]). T2DM undoubtedly comes with a financial burden due to the cost of necessary medications as we-ll as glucose monitoring, thereby making it more affordable to individuals with higher income levels. [21]. The current standard of SMBG routine uses glucometer, lancets and test strips, however standard guidelines for frequency of testing is yet available [31]. In 2012, the average cost per testing strips in the United States was $0.98 (1 USD = 4.4 MYR) [63]. A lower cost of SMBG was offered in this subscription model at a cost of MYR 0.72 per strip, and participants were recommended to test at least twice daily. Worth to take note that frequency of SMBG is associated with reduction of HbA1c level (Moström, et al., 2016).

In this study, participants in the intervention group demonstrated improved blood glucose management, and it is parallel to other studies in the attainment of HbA1c (%) level by using SMBG [12, 64]. Specifically, 0.4% mean reduction in HbA1c was documented in the final intervention stage, whilst the control group showed an increasing trend over the course of 6 months (Table 2). This reduction aligns with a meta-analysis study by Cunningham et al. [10], which reported improvements in HbA1c ranging from a 0.44% to 0.76% decline with self-management education for diabetes patients. The implementation of only standard T2DM management practice might be insufficient to advocate long term self-care and sustainability of motivation (Gunawardena, et al., 2018). Meanwhile, a randomized controlled trial proves that SMBG with a mobile management platform has significantly improves the proportion of patients who achieve adequate glycaemic control [64]. Therefore, this population is strongly recommended to imply SMBG and utilize various consultation media to ensure better treatment adherence [59].

The quality of life among T2DM patients have been studied by researchers locally [1, 28, 38]. Generally, T2DM patients have poorer quality of life when compared to those who are healthy and it has become an important measure in diabetes management since the treatment itself may influence patients’ physical and mental well-being [29]. Interestingly, this study found a significant change in one of SF-36 Mental Health domains, the Role-Emotional (Table 3). This domain assesses the limitations on routine activities due to emotional problems. Higher score indicates reduce limitation caused by emotional-related issues. Participants in both groups showed improvement over the 6 months period however the improvement is much enhanced in the intervention group.

In the present study, the subscription model itself remarks the existence of emotional support to the subscribers, where they received instant message through the mobile app based on their blood glucose level or whenever they needed assistance related to health issues. As reported by a randomized controlled trial, telecare has successfully improved quality of life and made T2DM patients more engaged with self-care and improved understanding in disease management [30]. The range and availability of mobile applications is expanding and supports empathy in gaining knowledge to better comprehend things such as targeting various goals in health matters [49]. Emotional responses were prominent in self-managing a disease and negligence would result in health deterioration [58]. Research has consistently documented the beneficial effects of emotional and social support on mental well-being particularly for diabetes patients [20, 45].

Further, we analyzed the association of blood glucose and SF-36 domain. The negative association of Physical Functioning and HbA1c remarks the importance of optimization of blood glucose control among T2DM patients. Physical functioning, which encompasses the ability to perform activities of daily living and engage in exercise or physical activities can influence person’s ability to maintain an active lifestyle [2]. Both physical functioning and HbA1c are important predictors of health outcomes and complications. Limitations in physical functioning can lead to decreased mobility and functional decline, further exacerbating health issues [61]. The growing evidence also suggest that T2DM patients with better blood glucose control have better physical functioning and signifies incorporation of self-care behaviour into life routines [11]. It is also possible that the association of this health domain may be attributed to the subscription program itself that motivates the participants to have a better understanding in diabetes management.

## Limitation

In the current study, the participants’ diet and physical activity were not considered. As these factors play crucial role in health outcomes of T2DM patients, it could limit the study’s comprehensiveness. Future intervention should incorporate a holistic approach and addressing these lifestyle elements. Additionally, this study did not separate the SMBG monitoring template according to mode of treatment as recommended by the established guidelines from Malaysian Clinical Practice Guidelines (CPG) (Ministry of Health, 2020) that might lead to disadvantages for regular blood glucose control. Apart from that, this study fails to show significant results in body weight management and other health-related quality of life measures over 6 months duration. The possible reason is due to small sample size and most results showed small effect size (*η* p2) values. Another reason could be that the results were confounded because the sampling technique utilized was not random. Selection bias arises since participants were not randomly assigned which leads to systematic differences between the groups. The higher income members were favoured because subscribing to the program came at a higher expense than opting out. Instead of just concentrating on the patient's health, the intervention's technologically savvy participants will gain more from the use of linked devices. Additionally, there is a risk of performance bias, wherein participants in the intervention group might exhibit greater motivation to adhere to diabetes management plan, potentially altering their behaviour compared to the control group.

A glucose monitoring subscription service, while offering convenience and advanced technology for managing diabetes, presents significant limitations in terms of socioeconomic equity. Subscriptions often require ongoing payments, which can be prohibitive for individuals with lower incomes or limited access to financial resources. This creates a disparity where those who can afford the subscription receive better diabetes management compared to those who cannot. In the future, studies should ensure the intervention is accessible and beneficial across diverse demographic groups. In the early stage, the researcher should tailor the intervention to address specific needs within the communities. Also, to address this issue, government intervention is crucial. By subsidizing the glucose monitoring program through public healthcare initiatives, the government can ensure equitable access to this vital technology for all diabetic patients. Subsidies could be targeted towards low-income individuals or those without adequate insurance coverage, effectively levelling the playing field and allowing everyone to benefit from advancements in diabetes care. Such initiatives not only promote health equity but also reduce the long-term healthcare burden associated with poorly managed diabetes. In summary, for more effective and comprehensive intervention, it is crucial to consider the equity from the outset and address this concern to positively impact a broader population, specifically T2DM patients.

## Conclusion

This preliminary study signifies the improvement of blood glucose control and health related quality of life among T2DM participants who joined a subscription model of SugO365 for SMBG. The optimization of SMBG and digitization of care combining standard and telemonitoring improved the adherence for self-care. The subscription model is feasible, useful, and has the potential to be implemented as an effective tool for diabetes care. Future studies should involve larger sample size with more outcome measures related to health such as lipid or renal profile with more intensify program and longer duration.

## Figures and Tables

**Fig. 3:**
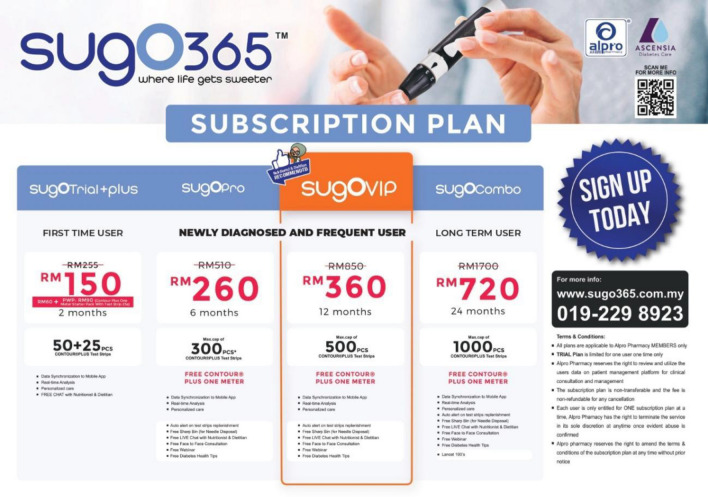
Sugo 365 Subscription Plans

**Fig. 4:**
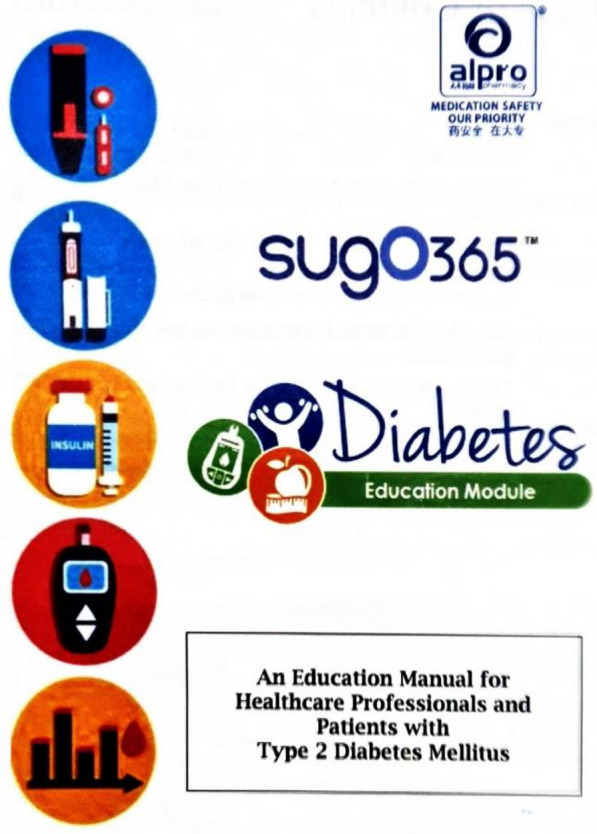
Sugo 365 Education Booklet

## Data Availability

The datasets supporting the conclusions of this article are included within the article and its additional files.

## Appendix

See Figs 3 ,4
